# Human *BDH2*, an anti-apoptosis factor, is a novel poor prognostic factor for *de novo* cytogenetically normal acute myeloid leukemia

**DOI:** 10.1186/1423-0127-20-58

**Published:** 2013-08-14

**Authors:** Wen-Chi Yang, Wan-Chi Tsai, Pai-Mei Lin, Ming-Yu Yang, Yi-Chang Liu, Chao-Sung Chang, Wen-Hui Yu, Sheng-Fung Lin

**Affiliations:** 1 Division of Hematology and Oncology, Department of Internal Medicine, Kaohsiung Medical University Hospital, No. 100 Tzyou 1st Road, 807 Kaohsiung, Taiwan; 2 Graduate Institute of Medicine, Kaohsiung Medical University, No. 100 Tzyou 1st Road, 807 Kaohsiung, Taiwan; 3 Department of Medical Laboratory Science and Biotechnology, Kaohsiung Medical University, No. 100 Tzyou 1st Road, 807 Kaohsiung, Taiwan; 4 Department of Nursing, I-Shou University, No. 1, Section 1, Syuecheng Road, Dashu Township, 824 Kaohsiung City, Taiwan; 5 Graduate Institute of Clinical Medical Sciences, College of Medicine, Chang Gung University, 59 Wen-Hwa 1st Road, Kwei-Shan 333 Tao-Yuan, Taiwan; 6 Faculty of Medicine, College of Medicine, Kaohsiung Medical University, No. 100 Tzyou 1st Road, 807 Kaohsiung, Taiwan

**Keywords:** BDH2, Survivin, Cytogenetically normal acute myeloid leukemia, Prognosis, Survival, Apoptosis

## Abstract

**Background:**

The relevance of recurrent molecular abnormalities in cytogenetically normal (CN) acute myeloid leukemia (AML) was recently acknowledged by the inclusion of molecular markers such as *NPM1*, *FLT3*, and *CEBPA* as a complement to cytogenetic information within both the World Health Organization and the European Leukemia Net classifications. Mitochondrial metabolism is different in cancer and normal cells. A novel cytosolic type 2-hydroxybutyrate dehydrogenase, BDH2, originally named DHRS6, plays a physiological role in the cytosolic utilization of ketone bodies, which can subsequently enter mitochondria and the tricarboxylic acid cycle. Moreover, BDH2 catalyzes the production of 2, 3-DHBA during enterobactin biosynthesis and participates in 24p3 (LCN2)-mediated iron transport and apoptosis.

**Results:**

We observed that *BDH2* expression is an independent poor prognostic factor for CN-AML, with an anti-apoptotic role. Patients with high *BDH2* expression have relatively shorter overall survival (*P* = 0.007) and a low complete response rate (*P* = 0.032). *BDH2*-knockdown (*BDH2*-KD) in THP1 and HL60 cells increased the apoptosis rate under reactive oxygen species stimulation. Decrease inducible survivin, a member of the inhibitors of apoptosis family, but not members of the Bcl-2 family, induced apoptosis via a caspase-3-independent pathway upon *BDH2*-KD.

**Conclusions:**

*BDH2* is a novel independent poor prognostic marker for CN-AML, with the role of anti-apoptosis, through surviving.

## Background

While morphological evaluation of bone marrow (BM) and blood remains a cornerstone for the diagnosis of acute myeloid leukemia (AML), the presence or absence of specific cytogenetic and molecular abnormalities is useful not only for determining overall prognosis but also for guiding treatment. Clonal chromosomal aberrations are not detected in 40–50% of patients [1]. Both the World Health Organization and the European Leukemia Net classifications have included molecular markers such as *NPM1*, *FLT3* and *CEBPA* as a prognostic factor for cytogenetically normal-acute myeloid leukemia (CN-AML) reinforcing their importance in cytogenetics [2,3]. Other mutated genes (e.g., *WT1, IDH1/IDH2, TET2, RUNX1,* and *MLL*) or aberrantly expressed ones (e.g., *BAALC, ERG, EVI1,* and *miR-181a*) will likely become useful in refining molecular risk in CN-AML [4-16]. Mithat Gönen et al. reported a panel of genetic profile that related to prognosis of AML [17].

In 1926, Otto Warburg initiated research on mitochondrial alterations in cancer and proposed a mechanism to explain the differences in energy metabolism between normal and cancer cells, suggesting that mitochondrial alterations could provide unique therapeutic targets in various cancer types [18]. Understanding the existing cross-talk between cellular metabolism and growth control has resulted in a better understanding of normal and disease processes and has facilitated the discovery of new treatment modalities in oncology, for example, trastuzumab and imatinib [19,20].

Guo et al. identified *BDH2* as a short-chain dehydrogenase/reductase family member, originally named as *DHRS6*[21] BDH2 is a novel cytosolic-type 2-hydroxybutyrate dehydrogenase and has a physiological role in the utilization of cytosolic ketone bodies, which can subsequently enter mitochondria and the tricarboxylic acid cycle [21]. Recently, Devireddy et al. reported that BDH2 catalyzes the production of 2,3-DHBA during enterobactin biosynthesis and participates in 24p3 (LCN2)-mediated iron transport and apoptosis [22]. We reported that *LCN2* expression is associated with prognosis in CN-AML previously [23]. While BDH2 is a gene related to mitochondria metabolism and participates in *LCN2* mediated iron transport and apoptosis, we have interest to know if *BDH2* has prognostic impact on CN-AML.

Here, we analyzed the relationship between *BDH2*, an enzyme related to the lipid metabolic pathway and iron transport, and CN-AML. We assessed whether *BDH2* is a prognostic factor for patient survival. Additionally, we investigated the mechanism underlying the prognostic ability of *BDH2* by using RNA interference-mediated knockdown of *BDH2* (BDH2-KD) in cell lines.

## Methods

### Ethics statement

This research was approved by Kaohsiung-Medical University Chung-Ho Memorial Hospital institutional review boards and ethics committees. All human participants provided written informed consent.

### Patients

We enrolled 130 patients newly diagnosed with CN-AML from August 2001 to February 2012, in a single medical center for retrospective analysis. Only 113 patients (including 49 female and 64 male patients) could be analyzed because of poor RNA quality in the remaining 17 samples. The average patient age was 54.5 years (age range, 22–86 years), with 47 patients more than 60 y/o. We also collected 43 normal BM samples, defined by normal BM findings in both BM aspiration smears and biopsy pathology reports and with no cytogenetic abnormalities, as a control group. Most patients were lymphoma without BM involvement. Moreover, we random collected bone marrow samples with good RNA quality from 10 newly diagnosed AML patients with AML-ETO fusion gene (translocation the AML1 [CBFA2, RUNX1] gene in the 21q22 region is fused to the ETO [MTG8 , RUNX1T] gene in the 8q22 region), 3 patients with cytogenetic finding of inv (16), as a good risk group, and 25 patients with multiple chromosomes abnormalities (more than 3 abnormalities) as a poor prognostic group from the samples in the past 10 years. Patients with chromosome 8 abnormality was excluded to avoid contaminated by myelodysplasia syndrome transformed AML.

Eighty-six CN-AML patients received conventional intensive induction chemotherapy consisting of 7 days of cytarabine at 200 mg·m^−2^·day^−1^ and 3 days of daunorubicin at 45 mg·m^−2^·day^−1^ (I3A7). Patients who failed to achieve complete remission (CR) but attained partial remission received the second induction chemotherapy with 70% doses of I3A7 under nadir status (between 7 and 10 days after first intensive chemotherapy). Patients achieving CR received consolidation chemotherapy with high dose AraC (cytarabine at 1 to 2 g·m^−2^·day^−1^ on day 1, 3 and 5). Based on the findings of these patients, we analyzed clinical outcomes, including overall survival (OS) and leukemia-free survival (LFS). Patients without CR after 2 rounds of intensive chemotherapy with good performance status (Eastern Cooperative Oncology Group, ECOG 0 or 1) and those with CR with a poor prognostic factor, including patients with delayed CR and *FLT3* internal tandem duplication (*FLT3*-ITD) mutation detected after 2011, received hematopoietic stem cell transplantation (HSCT).

### Clinical end points

CR was defined as recovery of morphologically normal BM and blood counts (i.e., neutrophil count ≥ 1.5 × 10^9^/L and platelet count ≥ 100 × 10^9^/L) and no circulating leukemic blasts or evidence of extramedullary leukemia. Relapse was defined by ≥5% BM blasts, circulating leukemic blasts, or development of extramedullary leukemia. OS was measured from the date of initial diagnosis until the date of death, censoring for patients alive at the last follow-up. LFS was measured from the date of CR until the date of relapse or death, regardless of the cause of death, censoring for patients alive at the last follow-up. Relapse-free survival (RFS) was measured from the date of diagnosis until the date of relapse or death.

Quantitative real-time reverse transcriptase-polymerase chain reaction (qRT-PCR) for mRNA expression analysis of *BDH2*, *LCN2*, *Meningionma1* (*MN1)*, *ETS-related gene (ERG)*, *micro-RNA-181a (miR-181a)*, and *micro-RNA-3151 (miR-3151).*

BM samples were collected at first diagnosis, and total RNA was extracted using the Trizol method (Invitrogen, Carlsbad, CA, USA). Furthermore, the same method was used to extract total RNA for RNA interference-mediated *BDH2*-KD in THP1 and HL60 cell lines. The RNA input (2 μg) for cDNA synthesis was determined by OD_260_ measurement, and cDNA was reverse transcribed using a TaqMan® High Capacity Reverse Transcription Kit (Applied Biosystems, Carlsbad, CA, USA) according to the manufacturer’s protocols. The cDNA sequences of *BDH2* and *LCN2* were evaluated, and the specific forward and reverse primers and TaqMan® probe were designed using Primer Express software version 1.5 (Applied Biosystems). The TaqMan® MGB probe designed by the software was synthesized and labeled with FAM fluorescent dye (Applied Biosystems). The mRNA expression levels of *BDH2* and *LCN2* were analyzed by qRT-PCR with the following primer sets and probes. *BDH2*: forward primer 5′-TTC CAG CGT CAA AGG AGT TGT-3′, reverse primer 5′-TTC CTG GGC ACA CAC AGT TG-3′, TaqMan® MGB probe 5′-ACA GAT GTG TGT GTA CAG CAC-3′ and *LCN2*: forward primer 5′-GGT ATG TGG TAG GCC TGG CA-3′, reverse primer 5′-AAC AGG ACG GAG GTG ACA TTG T-3′, TaqMan® MGB probe 5′-ACC CGC AAA AGA TGT A-3′. Expression of human *β-actin* was used to normalize *BDH2*, *LCN2*, *ERG* and *MN1*gene expression in qRT-PCR. Expression of human *U6 snRNA* was used to normalized *miRNA181a* and *miRNA3151* gene expression in qRT-PCR. This TaqMan® endogenous control and primers and TaqMan® probes of *ERG1*, *MN1*, *miRNA-181a* and *miRNA-3151* were purchased from Applied Biosystems. All reactions were carried out in a 25-μL final volume containing 200 ng of cDNA (as total input RNA), 400 nM of each primer, 200 nM of probe, and 12.5 μL of 2X TaqMan® Universal PCR Master Mix (Applied Biosystems).

For miRNA detection, RT reactions were performedwith 10 ng of total RNA, 50 nM stem–loop microRNA-specific RT primers, 1× RT buffer, 0.25 mM of dNTPs, 3.33 U/μl MultiScribe RTase and 0.25 U/μl RNase inhibitor. The reaction mixture was incubated for 30 min at 16°C and 30 min at 42°C, followed by 5 min incubation at 85°C to inactivate the RTase enzyme. RT products were subjected to microRNA expression assay for real-time quantitative PCR in a 20-μl final volume containing 2 μl of RT product, 1 μl of 20× TaqMan® micro-RNA Assay (Applied Biosystems), and 10 μl of 2× TaqMan® Universal PCR Master Mix (Applied Biosystems).

qRT-PCR was performed in an ABI Villi 7 Sequence Detector (Applied Biosystems), and the PCR cycling parameters were set as follows: 95°C for 10 min followed by 40 cycles of PCR reactions at 95°C for 20 seconds and 60°C for 1 min. The expression levels of the *BDH2* and *LCN2* genes were normalized to the internal control *β-actin* to obtain the relative threshold cycle (ΔC_T_). The relative expression between CN-AML and controls was calculated by the comparative C_T_ (ΔΔC_T_) method. The C_T_ values of *β-actin* were controlled between 18 and 22.

### Mutation analysis of *NPM1*, *FLT3, CEBPA, mixed lineage leukemia gene (MLL), IDH1/2* and *DNMT3A*

BM samples that were collected at diagnosis were retrospectively analyzed for gene mutations. Genomic DNA was extracted from mononuclear cell preparations using an Illustra™ blood genomicPrep Mini Spin Kit (GE Healthcare UK Limited, UK). The additional molecular markers associated with AML with normal karyotype, i.e., *FLT3*-ITD, *FLT3* tyrosine kinase domain (*FLT3*-TKD) mutation, *NPM1* mutation, *CEBPA* mutation, isocitrate dehydrogenase 1/2 (*IDH1/2*), DNA (cytosine-5)-methyltransferase 3A *(DNMT3A)* and *mixed lineage leukemia gene (MLL)* were screened as previously described [17,24-27].

PCR products were analyzed by agarose gel electrophoresis and purified using a QIAquick PCR-purification kit (Qiagen, Chatsworth, CA, USA). Purified PCR products were directly sequenced with the forward or reverse primers of each gene using an ABI BigDye Terminator Cycle Sequencing Kit (Applied Biosystems) in an ABI Prism 310 DNA sequencer (Applied Biosystems).

### Cell culture

The THP1 cell line, an acute myelomonocytic leukemia cell line, was cultured in RPMI medium (GIBCO, Life Technologies, Hong Kong) supplemented with 10% fetal bovine serum (FBS) (GIBCO), 1% penicillin/streptomycin (P/S), 4.5 g/L glucose, 10 mM HEPES (4-(2-hydroxyethyl)-1-piperazineethane-sulfonic acid), 1 mM sodium pyruvate, and 1% beta-mercaptoethanol. For THP1 cells infected with shRNA empty vector and shRNA-BDH2 lentivirus, 1 μg/mL puromycin (Sigma-Aldrich, USA) was added as a stress selector.

HL60 cells, an acute promyelocytic leukemia cell line with t(15;17), were cultured in IMDM medium (GIBCO) supplemented with 1.5 g/L sodium bicarbonate, 20% FBS, and 1% P/S. Puromycin (1 μg/mL) was added to select cells with RNA interference-mediated BDH2-KD.

All cells were cultured at 37°C in a humidified atmosphere containing 5% CO_2_. All native cell lines were purchased from Food Industry Research and Development Institute, Taiwan.

### RNA interference-mediated *BDH2*-KD in THP1 and HL60 cells

The shRNA-*BDH2* lentivirus particle was purchased from Sigma. The clones TRCN0000036735, 0000036736, 0000036738, and 00000244979 were identified as shRNA-*BDH2*-1, shRNA-*BDH2*-2, shRNA-*BDH2*-3, and shRNA-*BDH2*-4, respectively. Naive THP1 and HL60 cells were transduced with lentiviruses expressing shRNAs and selected for puromycin resistance (1 μg/mL). The knockdown efficiency was assessed by qRT-PCR and western blot analyses.

### Statistical analysis

We used software SPSS 17.0 for statistical analysis. ANOVA was used to compare the differences in *BDH2* mRNA expression and *LCN2* mRNA expression between patients with CN-AML, AML-ETO (+) under molecular studies, inv (16) under cytogenetic study and those with normal BM. Correlation regression was used for analysis if there was a correlation between *BDH2* and *LCN2* expression. We used the receiver operating characteristic (ROC) curve to estimate the cutoff point for *BDH2* to predict death in CN-AML patients.

Time-to-event analysis involved estimating the probability that an event would occur at different time points. The end-point of follow-up for patients who developed AML was the date of death and for those who were lost to follow-up was the date of the last visit, to arrive at “censored” data. Two-sample *t*-tests and *X*^*2*^ square tests were used to analyze the differences in age, sex, peripheral white blood cells (WBCs), hemoglobin (Hb), platelet and blast counts, CD34 and blasts percentage in BM, percentage of *FLT3-*ITD*, FLT3-*TKD*, NPM1* mutations, *CEBPA* mutation, *IDH1/2, DNMT3A* and *MLL* mutations in the *BDH2* low and high expression groups (*BDH2*^*low*^ and *BDH2*^*high*^, respectively) and younger and older patients group (separate at 60 year-old). ANOVA were used to analyze *ERG, NM1, miR-181a* and *miR-3151* in different groups of *BDH2* expression and age. We divided low and high expression of *BDH2* using the median BDH2 expression level (ΔC_T-*BDH2*_ = 9.0060), which was similar to the cutoff point from the ROC curve prediction (ΔC_T-*BDH2*_ = 9.0113). The Kaplan–Meier estimates were computed for the risk among different categories, were compared by Log-rank tests. The analysis was performed to estimate the differences in OS among patients with different *BDH* levels, and *FLT3*-ITD mutation as well as the differences in LFS and RFS between *BDH2*^*high*^ and *BDH2*^*low*^ groups.

Two sets of hazard rate ratios (HRR) were computed for analysis factors by cox regression analyses. The univariate HRRs were estimated from separate Cox regressions with one analysis factor at a time. The factors included all molecular analyses and other parameters. The multivariable-adjusted HRRs were computed from Cox regression with additional variables of the factors that were identified as being statistically significant in the univariate analysis.

### Apoptosis assay and flow cytometry

We treated THP1 and HL60 cells lines, including shRNA-*BDH2* infected, shRNA empty vector infected, and parental cells, with 50 μM H_2_O_2_ for 30 min and with 15 μM H_2_O_2_ for 30 min, respectively. Then apoptosis was examined using the ApoScreen™ Annexin V-FITC Apoptosis Kit (Beckman Coulter, CA, USA). Meanwhile, caspase-3 was also analyzed by flow cytometry in cells treated with H_2_O_2_ using FITC rabbit anti-active caspase-3 (BD Pharmingen, CA, USA).

### Western blot analysis

Cells were treated with 15 and 50 μM H_2_O_2_ for 2 h and were then lysed in cold lysis buffer supplemented with protease inhibitor cocktails (BioExpress, UT, USA, added at a 1:100 dilution). Cell lysates were separated by SDS-PAGE and transferred to a polyvinylidene fluoride membrane (PerkinElmer, Taiwan). Blots were probed with primary antibodies specific for the following proteins: PARP, caspase-3, survivin (Cell Signaling Technology, MA, USA), Bcl-xL, Bax, XIAP (Santa Cruz Biotechnology, Texas, USA), BDH (Sigma), and β-actin (Millipore Corporation, MA, USA). After incubation with the antibody, the proteins were detected with enhanced chemiluminescence (ECL, PerkinElmer).

### JC-1 mitochondrial membrane potential detection

The mitochondrial membrane potential was analyzed using a MitoProbe™ JC-1 Assay Kit (Life technologies, CA, USA). The collapse of the electrochemical gradient across the mitochondrial membrane was measured using a fluorescent cationic dye, JC-1. This dye exhibits potential-dependent accumulation in the mitochondrial matrix. Further, 1 × 10^6^ cells were incubated with 2 mM JC-1 at 37°C and 5% CO_2_ for 30 min. Cells were washed twice with PBS at 4°C, resuspended in 1 mL PBS, and assessed by a Beckman Coulter Epics XL.

## Results

### Association of *BDH2* expression with molecular markers and clinical characteristics

**Figure 1:**
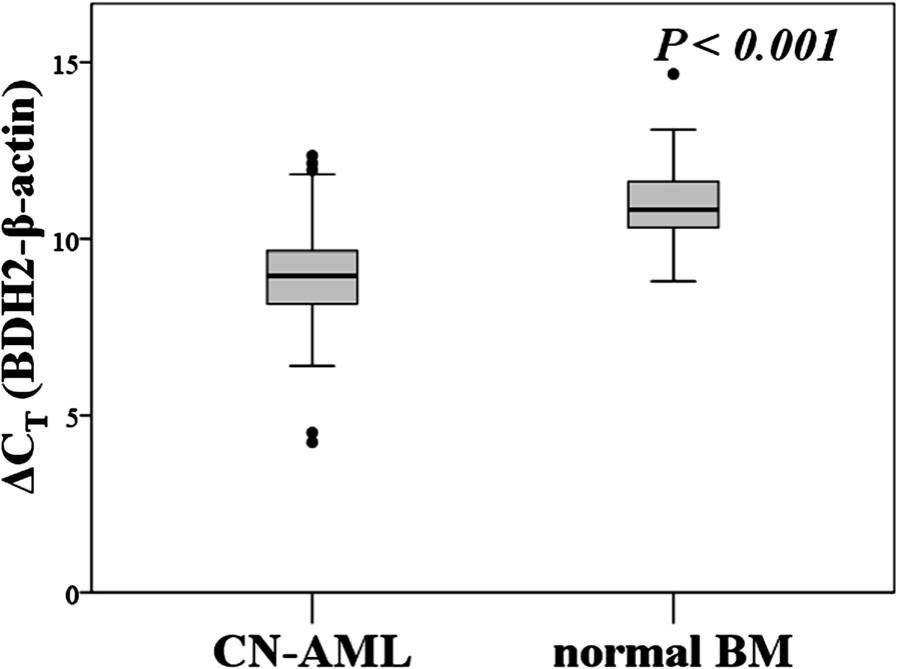
**mRNA expression levels of *BDH2 *in bone marrow.***BDH2*mRNA expression levels in bone marrow from patients with normal bone marrow and cytogenetically normal AML (CN-AML). *P<0.001.* Higher ΔC_T-*BDH2*_ means lower mRNA expression levels. BM: bone marrow. Sample number of bone marrows from normal bone marrow and CN-AML are 43 and 113.

The mRNA expression of *BDH2* was higher in patients with CN-AML and poor risk than in those with normal BM (*P* < 0.001, Figure 1 and Additional file 1: Figure S1). The mRNA expression of *LCN2* was lower in patients with CN-AML, inv (16) and poor risk group that we already reported [23]*.* However, we did not see difference of *BDH2* mRNA expression between good risk groups and normal bone marrow or CN-AML patients. There was no correlation between *BDH2* and *LCN2* expression (Additional file 2: Figure S2).

We focused on CN-AML patients and used the median *BDH2* mRNA expression level, ΔC_T-*BDH2*_ = 9.0060, as the cutoff point. This value is the same as the cutoff point predicted by the ROC curve. Among the 113 patients newly diagnosed with CN-AML, no differences were observed between *BDH2*^*high*^ and *BDH2*^*low*^ groups with regard to clinical features or biological characteristics such as age, sex, WBCs, Hb, platelets, blasts in peripheral blood, blasts in BM, amount of CD34 expression in BM myeloblasts, and French-American-British (FAB) classification subtypes (Table 1). Moreover, no differences were observed with regard to these clinical features between the 2 groups, among the 86 patients with CN-AML with intensive induction chemotherapy (data not shown).

**Table 1:** **Comparison of clinical manifestations between patients with AML in low and high *BDH2 *expression groups**^**a**^

**Variables**	**Total (n = 113)**	**Low *BDH2, *expression, (n = 57)**	**High *BDH2, *expression, (n = 56)**	***P***
Sex^b^	113	57	56	*0.467*
Male	64	23	26	
Female	49	34	30	
Age (years)^c^	54.5 (21–86)	48 (22–86)	57 (21–85)	*0.193*
Laboratory data^c^				
WBC, ìL^−1^	30,830	37,615	24,370 (600–243,290)	*0.452*
	(300–296,300)	(300–216,820)		
Hb, g/dL	8.3	8.3	8.4	*0.365*
	(4.2–15.6)	(4.4–14.3)	(4.2–15.6)	
Platelet, ×1,000/ìL	42	44.5	41	*0.582*
	(3–369)	(4–175)	(3–369)	
Blast, ìL^−1^	20,188.5	13,074.15	26,001	*0.172*
	(0–287,411)	(126–145,572.7)	(0–287,411)	
Blast in BM, %	74.8% (24.8%–97.4%)	70.8% (24.8%–94.7%)	79% (27.2%–94.4%)	*0.424*
CD34 (+) in BM, %	31.1%	29.5%	33.25% (0%–99.5%)	*0.272*
	(0%–99.9%)	(0%–99.9%)		
FAB^b^				
M0	3	1 (1.75%)	2 (2.44%)	*0.057*
M1	28	8 (13.89%)	20 (43.90%)	
M2	47	25 (47.23%)	22 (34.15%)	
M3	0	0 (0%)	0 (0%)	
M4	23	15 (23.61%)	9 (14.63%)	
M5	7	6 (9.72%)	1 (0%)	
M6	0	0 (0%)	0 (0%)	
M7	4	2 (2.78%)	2 (4.88%)	
Undetermined	0	0 (0%)	0 (0%)	
Induction response^bd^				
CR	61 (70.93%)	37 (80.42%)^f^	24 (60%)^g^	0.032*
PR and Refractory		32	20	
Induction death		1	0	
Reach CR time^e^				
	38 ± 38.0	40.5 ± 44.3	36 ± 29.7	*0.526*

^a^The median value of *BDH2* expression in the total population was used as the cutoff level of 9.006 to define low- and high-expression groups.

^b^Number of patients (%).

^c^Median (range).

^d^Only the 86 patients who received conventional intensive induction chemotherapy, and then consolidation chemotherapy if CR was achieved, were included in the analyses.

^e^Patients received conventional intensive induction chemotherapy and achieved CR, median, days ± S.D.

^f^Only 46 patients received I3A7.

^g^Only 40 patients received I3A7 (Idarubicin and Ara-C).

*Statistically significant (*P* < 0.05).

The incidences of common genetic alterations in the *BDH2*^*high*^ and *BDH2*^*low*^ groups are shown in Table 2. On the whole cohort analysis, our patients showed similar incidences of *FLT3*-ITD and *FLT3*-TKD mutations when compared with data from Taiwan National University [28]; however, the incidences of *NPM1, MLL* and *CEBPA* mutations were higher and the incidence of *IDH1* mutation was lower. *FLT3*-ITD showed a higher mutation rate in the *BDH2*^*high*^ group (*P* = 0.030, Table 2) and *DNMT3A* showed a higher mutation rate in the *BDH2*^*low*^ group (*P* = 0.009, Table 2). We did not observe differences in *NPM1, FLT3-TD*, *CEBPA,* and *IDH1/2* mutations between the 2 groups (Table 2).

**Table 2:** **Comparison of other genetic alterations between AML patients in low and high *BDH2 *expression groups**

**Variant**	**Number of patients with the gene alternation (percentage)**			***P***
	**Whole cohort**	**Low *BDH2, *expression**	**High *BDH2, *expression**	
*NPM1*^mut^	36 (31.85%)	16 (28.07%)	20 (35.71%)	*0.271*
*FLT3-*ITD	23 (20.35%)	7 (12.28%)	16 (28.57%)	0.030*
*FLT3-*TKD	8 (7.08%)	5 (8.77%)	3 (5.36%)	*0.338*
*NPM1*^mut^/*FLT3-*ITD^neg^	20 (17.70%)	11(19.30%)	9 (16.07%)	*0.368*
*CEBPA*^a^	34 (30.09%)	16 (28.07%)	18 (32.14%)	*0.444*
*CEBPA*^Double mutation^	9 (7.96%)	3 (5.26%)	6 (10.71%)	*0.593*
*IDH1*^b^	3 (3.75 %)	1 (2.56 %)	2 (4.88%)	*0.592*
*IDH2*^b^	8 (10 %)	3 (7.69%)	5 (12.20%)	*0.508*
*DNMT3A*^b^	12 (15 %)	10 (25.641%)	2 (4.88%)	0.009*
*MLL*^b^	7 (8.75%)	3 (7.69%)	4 (9.76%)	*0.426*
*ERG*^c^	11.17 (10.26-12.08)	11.60 (10.48-12.72)	10.68 (9.17-12.20)	*0.320*
*MN1*^c^	12.98 (12.28-13.68)	13.35 (12.42-14.28)	12.55 (11.47-13.64)	*0.257*
*miR-181a*^c^	3.12 (2.57-3.67)	3.17 (2.35-3.98)	3.07 (2.30-3.84)	*0.864*
*miR-3151*^c^	12.35 (11.90-12.80)	12.18 (11.53-12.84)	12.53 (11.89-13.16)	*0.448*

Values are number (%) of patients with alteration.

^a^*CEBPA*^single and double mutations^.

^b^Only 80 patients with high quality of DNA to sequence; 39 patients are low BDH2 expression and 41 patients are high expression.

^c^Mean (95% CI).

*Statistically significant (*P* < 0.05).

### Gene alternations frequencies between younger and elder patients

As shown in Table 3, the frequency of *FLT3*-TKD mutation is higher in patients more than 60 years-old. And the *CEBPA* double mutation rate is higher in younger patients group. There are no different of *NPM1, FLT3*-ITD, *IDH1/2*, *DNMT3A* and *MLL* gene mutations, and no difference in *BDH2, ERG, MN1, miR-181a* and *miR-3151* expression levels, between different age group.

**Table 3:** **Comparison of other genetic alterations between older and younger AML patients**^**a**^

**Variant**	**Number of patients with the gene mutation (percentage); Median of RNA expression (delta C**_**T**_**)**			***P***
	**Whole cohort**	**60 years or younger, (n=69)**	**Older than 60 y/o, (n=44)**	
*NPM1*^mut^	38 (33.33%)	21 (30%)	17 (38.64%)	*0.227*
*FLT3-*ITD	23 (20.18%)	15 (21/43%)	8 (18.18%)	*0.433*
*FLT3-*TKD	8 (7.02%)	2 (2.86%)	6 (13.64%)	0.036*
*NPM1*^mut^/*FLT3-*ITD^neg^	22 (19.30%)	11(15.71%)	11 (25%)	*0.164*
*CEBPA*^a^	34 (30.36%)	18 (26.09%)	16 (37.20%)	*0.151*
*CEBPA*^Double mutation,b^	9 (78.04%)	8 (11.59%)	1 (2.23%)	0.016*
*IDH1*^c^	3 (3.75 %)	2 (4%)	1 (3.3%)	*0.686*
*IDH2*^c^	8 (10 %)	4 (8%)	4 (13.2%)	*0.454*
*DNMT3A*^c^	12 (15 %)	10 (25.641%)	2 (4.88%)	*0.095*
*MLL*	7 (8.75%)	4 (8%)	3 (10%)	*0.528*
*Delta BDH2*^d^	9.006 (4.53-12.36)	9.051 (4.53-12.36)	8.915 (6.4-12.15)	*0.102*
*ERG*^d^	10.595 (4.52-19.92)	10.88 (5.17-19.92)	9.74 (4.52-19.85)	*0.435*
*MN1*^d^	13.25 (7.75-19.87)	13.06 (7.76-19.87)	13.25 (7.75-18.38)	*0.992*
*miR-181a*^d^	3.21 (−3.8-8.44)	3.3 (−0.03-8.44)	2.49 (−3.8-8.2)	*0.140*
*miR-3151*^d^	12.685 (4.97-15.29)	12.685 (9.16-15.07)	12.6 (4.97-15.29)	*0.247*

Values are number (%) of patients with alteration.

^a^*CEBPA*^single and double mutations^.

^b^*CEBPA*^double mutation^*vs CEBPA*^single mutation^*vs* no mutation.

^c^Only 80 patients with high quality of DNA to sequence; 50 patients are 60 y/o or younger patients and 30 patients are high expression.

^d^Median *delta C*_*T*(_range).

*Statistically significant (*P* < 0.05).

### *BDH2* expression as a prognostic marker

**Figure 2:**
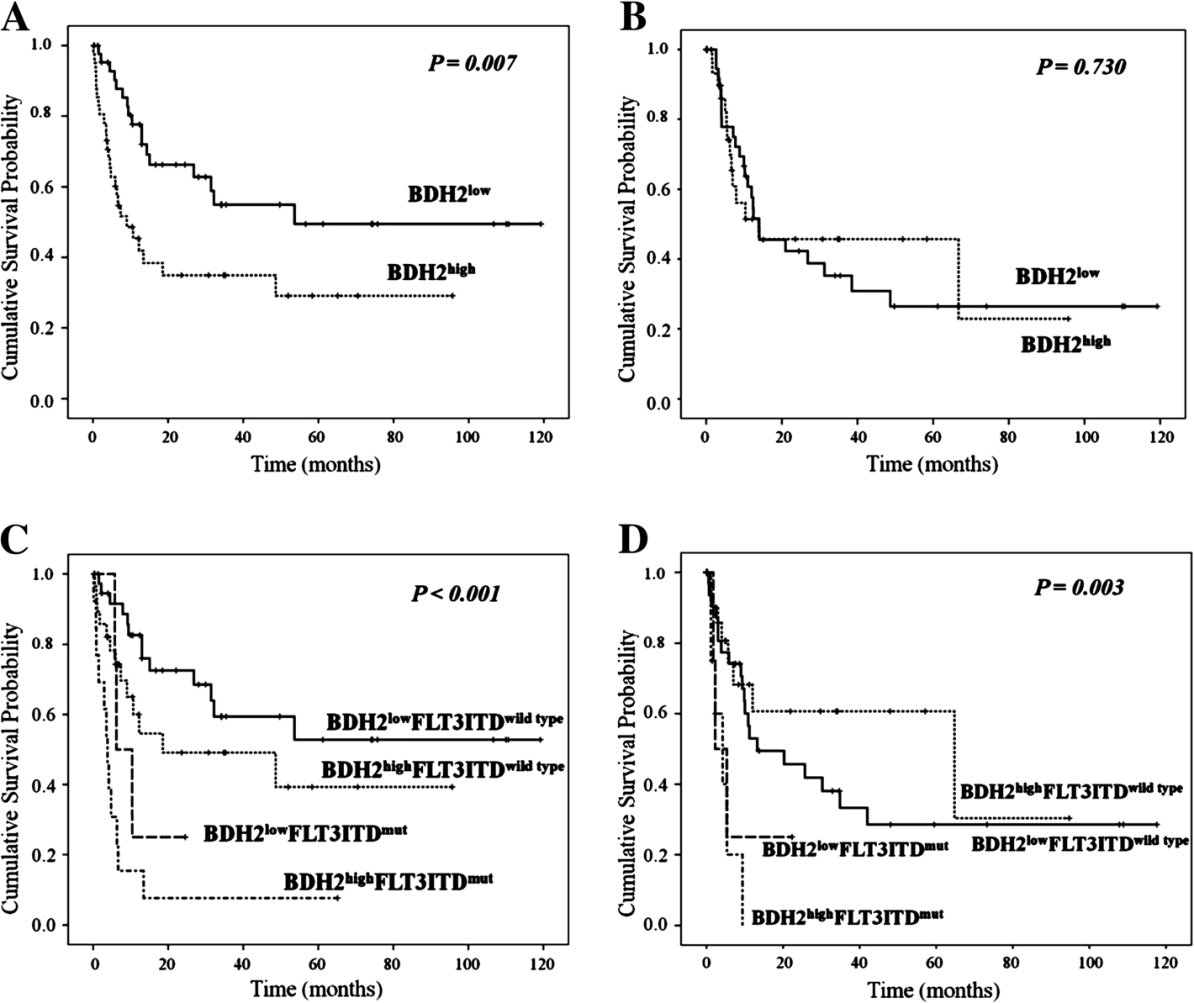
**Outcome of patients with CN-AML according to *BDH2 *expression levels and *FLT3-*ITD mutation status.** In 85 patients receiving intensive induction chemotherapy, we analyzed **A)** overall survival; **B)** leukemia-free survival between 2 different *BDH2* expression groups; and **C)** overall survival; **D)** leukemia-free survival between groups differentiated by *BDH2* expression level, combined with the *FLT3*-ITD mutation status.

We analyzed 86 patients who received a standard intensive chemotherapy. In response rate analysis, patients in the *BDH2*^*high*^ group showed a lower complete response rate (60%) than those in the *BDH2*^*low*^ group (80.42%). However, no difference was observed between the 2 groups with respect to the time required to reach a complete response (Table 1). We also analyzed complete response (CR) rate based on genetic alterations and noticed that patients with *DNMT3A* mutations had significant higher CR rate than patients without *DNMT3A* mutation (*P* = 0.012). We did not find significant difference in CR rate between *FLT3*-ITD, *NPM1*, *CEBPA* and *IDH1/2* mutations (data not shown). Results of the survival analysis showed that patients in the *BDH2*^*high*^ group had a lower overall survival (OS) with a medium survival of 9 months than those in the *BDH2*^*low*^ group with a median survival of 53.667 months (*P* = 0.007, Figure 2A). However, we did not note any difference in the LFS rates between the *BDH2*^*high*^ and *BDH2*^*low*^ groups, with median survivals of 12.033 months and 13.2 months, respectively (*P* = 0.730, Figure 2B).

In univariate analysis of the impact factors on OS, old age, high *BDH2* expression, and *FLT3*-ITD mutation adversely affected OS with statistical significance (*P* = 0.001, 0.007, and < 0.001, respectively; Table 4). *NPM1*, *FLT2*-TKD, *CEBPA*, *IDH1/2, DNMT3A* and *MLL* mutations did not show impact on OS. Multivariate analysis showed that *FLT3*-ITD, *FLT3*-TKD and *MN1* mutations adversely affected RFS. While *NPM1* mutation showed positive impact on RFS. High *BDH2* expression had a mild adverse effect on RFS without statistical significance. However, a significant, independent adverse impact of high *BDH2* expression, *FLT3-*ITD mutation, *MN1* mutation, and old age were observed in the OS multivariate analysis (Table 5). In patients with 60 year-old or younger, *FLT3*-ITD, *FLT3*-TKD, *MN1* mutations, and *ERG* and *BDH2* higher expression level showed adverse impact on survival. In elder patients, we saw *ERG* and *BDH2* higher expression level had poor survival (Table 6). No patients in elderly group had *DNMT3A* mutation.

**Table 4:** Univariate analyses of the impact on overall survival in patients with CN-AML who received intensive induction chemotherapy

**Variable**	**No. of patients**	**Overall survival**	
		**Median**^**a**^	***P***
Age, years			0.001^*^
60 or younger	66	32.2	
Older than 60	22	4.5	
WBC			*0.469*
50000/uL or less	49	31.433	
Greater than 50000/uL	37	13	
*BDH2*			0.007^*^
Lower expression	46	53.667	
Higher expression	40	9	
*CEBPA*			*0.755*
Double mutation	9	31.433	
Single mutation	16	53.667	
No mutation	61	16.267	
*NPM1*			*0.179*
Mutated	28	10.4	
Wild	58	32.2	
*FLT3-*ITD^*-*^			<0.001^*^
Mutated	17	4.767	
Wild	70	48.633	
*FLT3-*TKD			*0.596*
Mutated	7	13.00	
Wild	79	18.567	
*NPM1/FLT3-*ITD			*0.314*
*NPM1*^*+*^*/FLT3-*ITD^*-*^	15	NR	
Others	71	15.067	
*IDH1*^b^			*0.796*
Mutated	3	22.21	
Wild	61	27.055	
*IDH2*^b^			*0.749*
Mutated	4	21.91	
Wild	60	27.16	
*DNMT3A*^b^			*0.508*
Mutated	11	32.58	
Wild	53	25.63	
*MLL*^b^			*0.178*
Mutated	5	12.63	
Wild	59	13.12	
*BDH2*^*low*^*/FLT3-ITD*			<0.001^*^
*BDH2*^*low*^*/FLT3-ITD*^*wild type*^	40	722.16	
*BDH2*^*low*^*/FLT3-ITD*^*mutation*^	5	519.73	
*BDH2*^*high*^*/FLT3-ITD*^*wild type*^	27	371.56	
*BDH2*^*high*^*/FLT3-ITD*^*mutation*^	13	182.27	

Abbreviation: NR indicates not reached.

^a^Median, months.

^b^Only 64 patients who had high quality DNA preserved for sequence received standard intensive induction chemotherapy.

*Statistically significant (*P* < 0.05).

**Table 5:** **Multivariate analyses (cox regression) of relapse-free survival and overall survival**^**a**^

**Variables**	**Relapse-free survival**				**Overall survival**			
		**95% CI**				**95% CI**		
	**HR**	**Lower**	**Upper**	***P***	**HR**	**Lower**	**Upper**	***P***
Whole cohort (n = 86)								
Age^b^	3.083	0.184	51.762	*0.434*	3.252	1.322	8.002	0.010*
*NPM1*^c^	0.087	0.012	0.604	0.014*	0.392	0.128	1.195	*0.100*
*FLT3-*ITD^d^	21.079	1.427	311.302	0.026*	4.532	1.395	14.723	0.012*
*FLT3-*TKD	19.630	1.539	250.445	0.022*	1.801	0.184	17.603	*0.613*
*CEBPA*^e^	0.408	0.144	1.154	*0.091*	0.398	0.147	1.080	*0.070*
*IDH1*^c^	0.448	0.039	5.167	*0.520*	0.648	0.128	3.268	*0.599*
*IDH2*^c^	0.583	0.025	13.640	*0.737*	0.254	0.036	1.781	*0.168*
*DNMT3A*^c^	0.292	0.041	2.098	*0.221*	0.731	0.158	3.380	*0.689*
*MLL*^c^	31.310	0.729	1344.29	*0.073*	1.040	0.215	5.020	*0.961*
*MN1*	1.391	1.027	1.883	0.033*	1.195	1.025	1.394	0.023*
*ERG*	5.799	0.548	61.325	*0.144*	2.545	0.993	6.520	*0.052*
*miR-181a*	0.824	0.598	1.135	*0.236*	0.860	0.692	1.069	*0.174*
*miR-3151*	1.183	0.750	1.867	*0.469*	1.285	0.884	1.866	*0.189*
*BDH2*^f^	2.035	0.472	8.782	*0.341*	2.547	1.094	6.685	0.050*

Abbreviations: HR indicates Hazard ratio; CI, confidence interval.

^a^Only patients with intensive induction chemotherapy enrolled.

^b^Age > 60 relative to Age ≤ 60 (the reference).

^c^*NPM1*^mut^ versus *NPM1*^wild type^; *ID*H1^mut^ versus *IDH1*^wild type^; *IDH2*^mut^ versus *IDH2*^wild type^; *DNMT3A*^mut^ versus *DNMT3A*^wild^ type ; *MLL*^***mut***^ versus *MLL*^***wild***^*.*

^d^*FLT3-*ITD^mut^ versus *FLT3-*ITD^neg^.

^e^*CEBPA*^double-mutation^ versus *CEBPA*^single mutation^ versus *CEBPA*^no mutation^.

^f^*BDH2*^*high*^ relative to *BDH2*^*low*^ group.

*Statistically significant (P < 0.05).

**Table 6:** **Multivariate analyses (cox regression) of overall survival**^**a **^**in younger and older patients**

**Variables**	**Patients ≦ 60 y/o**				**Patients > 60 y/o**			
		**95% CI**				**95% CI**		
	**HR**	**Lower**	**Upper**	***P***	**HR**	**Lower**	**Upper**	***P***
*NPM1*^b^	0.000	0.000	1.490E89	*0.898*	0.263	0.021	3.377	*0.305*
*FLT3-*ITD^c^	7.505	1.538	36.614	0.013*	3.235	0.424	24.699	*0.258*
*FLT3-*TKD	9765.104	32.249	2.95E6	0.002*	144.617	0.000	3.75E169	*0.980*
*CEBPA*^d^	0.357	0.125	1.020	*0.054*	1.090	0.292	4.066	*0.898*
*IDH1*^b^	14220.871	0.000	3.9E99	*0.932*	174.990	0.000	4.56E169	*0.979*
*IDH2*^b^	0.000	0.000	2.695E210	*0.958*	0.459	0.050	4.242	*0.492*
*DNMT3A*^b^	146.313	0.000	4.16E97	*0.965*				
*MLL*^b^	0.059	0.000	1.78E94	*0.980*	2.245	0.173	29.122	*0.536*
*MN1*^e^	1.923	1.130	3.273	0.016*	1.198	0.916	1.567	*0.187*
*ERG*	53.777	1.820	1589.377	0.021*	32.349	2.889	362.237	0.005*
*miR-181a*	0.514	0.260	1.014	*0.055*	1.361	0.933	1.986	*0.110*
*miR-3151*	1.164	0.580	2.337	*0.669*	0.423	0.191	0.935	0.033*
*BDH2*^e^	4.829	1.002	23.274	0.050*	18.937	1.571	228.308	0.021*

*Abbreviations*: HR indicates Hazard ratio; CI, confidence interval.

^a^Only patients with intensive induction chemotherapy enrolled.

^b^*NPM1*^mut^ versus *NPM1*^wild type^; *ID*H1^mut^ versus *IDH1*^wild type^; *IDH2*^mut^ versus *IDH2*^wild type^; *DNMT3A*^mut^ versus *DNMT3A*^wild^ type.

^c^*FLT3-*ITD^mut^ versus *FLT3-*ITD^neg^.

^d^*CEBPA*^double-mutation^ versus *CEBPA*^single mutation^ versus *CEBPA*^no mutation^.

^e^*BDH2*^*high*^ relative to *BDH2*^*low*^ group.

*Statistically significant (P < 0.05).

By combining two independent prognostic factors, *BDH2* expression and *FLT3*-ITD mutation, we found that patients with *BDH2*^*low*^*FLT3*-ITD*-*wild type had the highest OS, with a median survival surpassing 10 years. On the other hand, patients with *BDH2*^*high*^*FLT3*-ITD-mutation had the worst overall survival, with a median survival of 3.833 months. We observed significant differences in the overall survival between the *BDH2* expression groups with and without the *FLT3*-ITD mutation (median survival: *BDH2*^*low*^*FLT3*-ITD-wild type vs. *BDH2*^*high*^*FLT3*-ITD-wild type vs. *BDH2*^*low*^*FLT3*-ITD-mutation vs. *BDH2*^*high*^*FLT3*-ITD-mutation *=* not reached vs. 18.567 months vs. 6.2 months vs. 3.833 months, *P* < 0.001, Figure 2C and 2D).

We analyzed 22 patients who received allogeneic hematopoietic stem cell transplantation, including 14 patients in the *BDH2* lower expression group and 8 in the *BDH2* higher expression group*.* Though there was no statistically significant difference, we saw a trend of longer survival from hematopoietic stem cell infusion in the *BDH2*^*low*^ group (*P* = 0.520, Additional file 3: Figure S3).

### Survivin reduction was responsible for inducing apoptosis in *BDH2-KD* cells under hyperoxidative stress via a caspase-3-independent pathway

**Figure 3:**
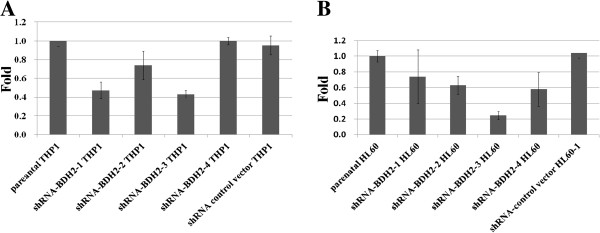
**shRNA interference-mediated knockdown efficiency in leukemia cell lines. ***BDH2* mRNA expression level in *BDH2*-knockdown, control vector infected, and parental cells. **A)** THP1 cells and **B)** HL60 cells, as determined by qRT-PCR analyses.

**Figure 4:**
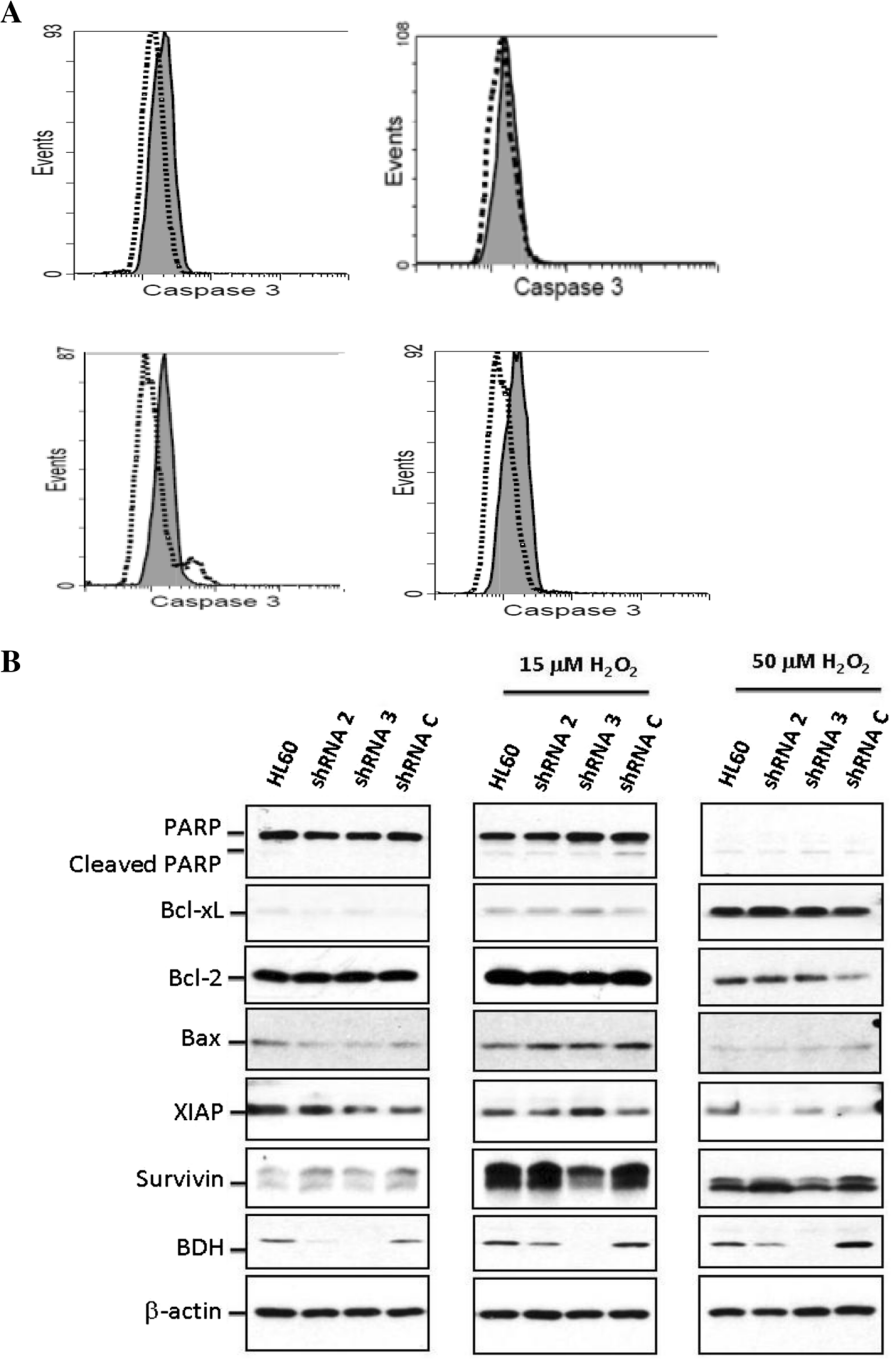
**Caspase-3 activity after treatment with 50 μM H**_**2**_**O**_**2 **_**for 2 h in the HL60 cell line. A)** Cleaved caspase-3 detected by flow cytometry. The gray area represents cells that were not subjected to the H_2_O_2_ treatment, and the dot- lines denote cells treated with H_2_O_2_. Top left: parental HL60; top right: shRNA-*BDH2*-2 HL60; bottom left: shRNA-*BDH2*-3 HL60; bottom right: shRNA control vector HL60. **B)** Bcl-2, Bcl-xL, BAX, PARP, survivin, XIAP, and BDH2 protein expression on a western blot analyses.

**Figure 5:**
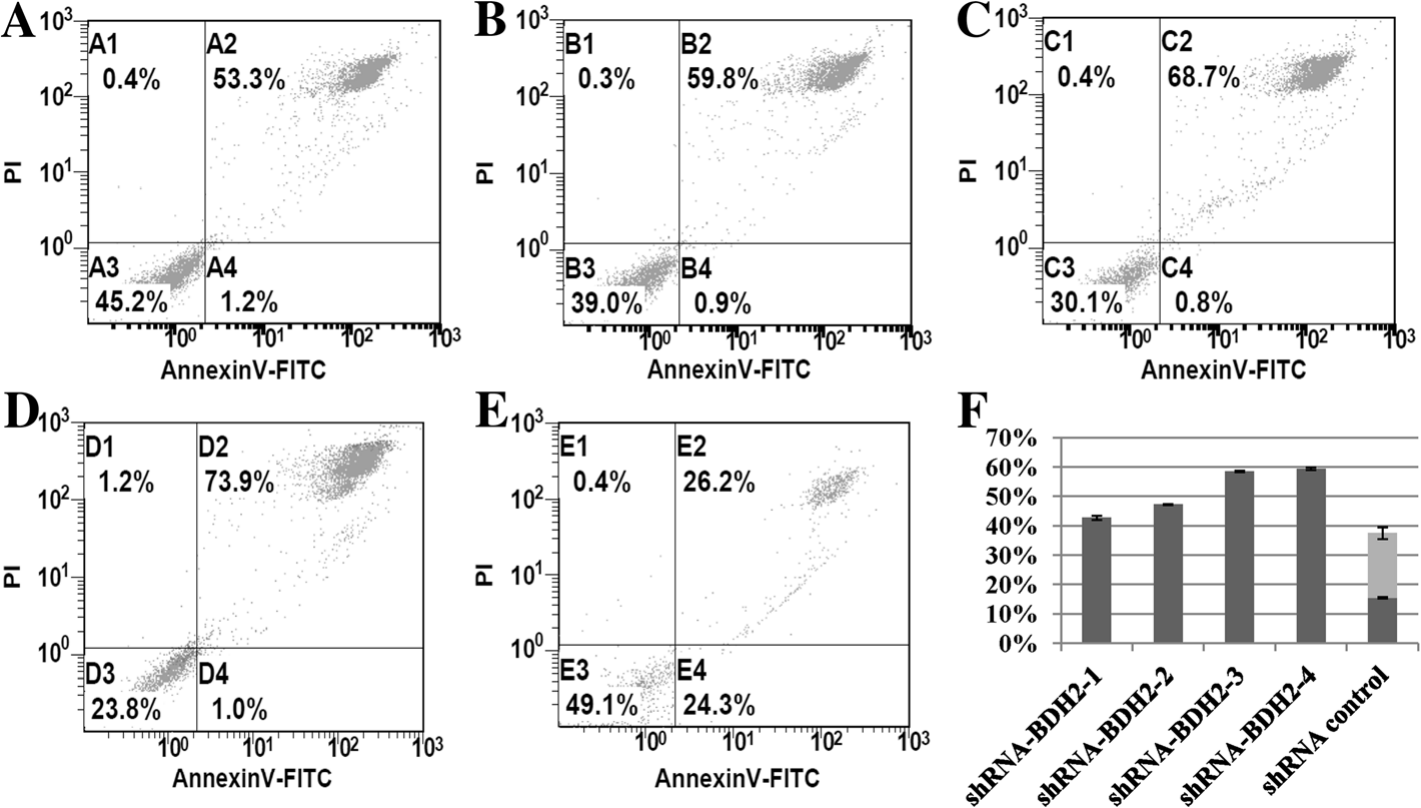
**Apoptosis rate in HL60 cells after treatment with 50 μM H**_**2**_**O**_**2 **_**for 2 h.** The apoptosis rate was dependent on the *BDH2-*knockdown efficiency: **A)** shRNA-*BDH2*-1 HL60; **B)** shRNA-*BDH2*-2 HL60; **C)** shRNA-*BDH2*-3 HL60; **D)** shRNA-*BDH2*-4 HL60; and **E)** shRNA control vector HL60; **F)** the apoptosis rate of each cell lines. Dark gray: late apoptosis bar; light gray bar: early apoptosis.

Reactive oxygen species (ROS) can induce apoptosis [29]. Devireddy et al. showed that the apoptosis rate increased in *BDH2*-KD FL5.12 cells upon H_2_O_2_ treatment [22]. The impact of *BDH2* was evaluated by using RNA interference-mediated *BDH2*-KD in THP1 and HL60 leukemia cell lines. The efficacy of *BDH2*-KD was confirmed at both RNA (Figure 3A and 3B) and protein levels (Figure 4B). Microscopic analysis under hyperoxidative stress showed that shRNA-*BDH2*-3 HL60 had more apoptotic cells (Additional file 4: Figure S4). This result was confirmed by Annexin V/PI staining analysis. Figure 5 shows the apoptotic population in different levels of *BDH2*-KD HL60 cells. After H_2_O_2_ treatment, *BDH2*-KD HL60 cells showed a more prominent increase in the secondary and forth quadrant, indicating that *BDH2* exerted an anti-apoptotic effect (Figure 5). The same effect was also observed in *BDH2*-KD THP1 cells (data not shown). Nevertheless, the conventional apoptosis-related proteins, including PARP, caspase-3, Bcl-xL, Bcl-2 and Bax, did not mediate H_2_O_2_-induced apoptosis in *BDH2*-KD HL60 cells (Figure 4B). Western blotting data also failed to show caspase-3 activation using flow cytometry analysis (Figure 4A), regardless of whether the expression level of BDH2 was high or low. Taken together, the results did not show a correlation between *BDH2*-KD and the activation of the Bcl-2/Bcl-xL-mediated intrinsic pathway via the caspase cascade to trigger apoptosis. This phenomenon correlated with mitochondrial membrane potential analysis (Additional file 5: Figure S5).

There is a family of functionally and structurally related proteins that serve as endogenous inhibitors of apoptosis (IAP) [30]. Survivin, the smallest member of the IAP family, increases during ROS stimulation to protect cells from apoptosis [30,31]. A marked induction of survivin upon H_2_O_2_ treatment was observed in our study, whereas XIAP was not altered in cells with different *BDH2* expression levels. Of note, the induction of survivin was abrogated in shRNA-*BDH2*-3 HL60 cells. These data suggest that survivin is responsible for elevating the apoptosis rate upon ROS treatment in *BDH2*-KD cells.

## Discussion

The prognosis of CN-AML depends on the molecular status (e.g., *FLT3, NPM1, CEBPA, IDH1/2, DNMT3A, MLL, ERG, MN1* and *micro-RNA-181a* and *3151)*; however, not all candidate markers have been detected so far. Energy metabolism differs in normal cells and cancer cells. Inborn errors of metabolism can induce cancer development [32]. Cancer can also result from deficiency or overactivity of enzymes, deficiency of a cofactor required for enzymatic activity, an abnormality in degradation or transport processes that lead to the accumulation of upstream metabolites, deficiency of a downstream metabolite, or diversion of the affected metabolic flux to secondary pathways [33].

There are 3 different types of insults related to the inborn errors of metabolism: (1) toxic accumulation of metabolites, (2) metabolite channeling, and (3) mitochondrial dysfunction. Toxin accumulation leads to an increase in oxidative stress and ROS, as seen with iron accumulation in hemochromatosis [34]. In addition, accumulation of toxic metabolites could affect gene expression or cause a shift to alternative metabolic pathways, which could lead to tumorigenesis [29].

Human *BDH2* (*DHRS6*) is an enzyme that participates in the citric acid cycle metabolism and ketogenesis [32], which may play a crucial role in promoting tumorigenesis [33,34]. In our results, *BDH2* mRNA expression was higher in the BM of patients with CN-AML and AML with multiple chromosome abnormalities, compared with normal BM samples. We focused on CN-AML patients. No differences were observed in the incidence of clinical pictures, including blast amount in BM, FAB classification, and alterations in genes, except *FLT3*-ITD and *DNMT3A*, between *BDH2*^*high*^ and *BDH2*^*low*^ groups. High *BDH2* expression is an independent indicator of poor prognosis of CN-AML, which may be related to a poor response to conventional intensive chemotherapy and a low CR rate. Given the independent adverse impact on survival and the low response rate in patients in the *BDH2*^*high*^ group in both younger and elder patients, we suggest that higher *BDH2* expression makes patients resistant to intensive induction chemotherapy. Although high *BDH2* expression does not shorten the duration of relapse once patients obtain CR, patients with low *BDH2* expression may benefit from further treatment, including chemotherapy and allogenetic hematopoietic stem cell transplantation, which may prolong survival time.

In outcome analysis by gene alternations, *FLT3*-ITD and *MN1* mutations, and *BDH2* were independent adverse prognostic factor for survival, with statistics significant. *ERG* and *MLL* mutations, and higher *miR-3151* expression level showed a trend of adverse impact on survival. *NPM1, CEBPA* mutations and *miR-181a* showed positive outcome in CN-AML patients. However, *DNMT3A* mutations showed a good impact on survival, that could not compatible with previous reports. The mutations in *DNMT3A* in eleven of twelve of our patients are R882 (4279129C->T). One patient with *DNMT3A* mutation is R882 (4279073G->T). Only 12 CN-AML patients have *DNMT3A* mutation. Because of small number of patients, we cannot say that *DNMT3A* mutations had positive impact in overall survival (OS) in our patients. And that is the reasons of non-significant impact of other well-known genes alternations in CN-AML in our study.

To test if *BDH2* can induce chemoresistance, we generated *BDH2*-KD leukemia cell lines. Oxidative damage is thought to be an important mechanism by which agents such as alkylators can damage DNA [35]. The intensive chemotherapy agents we used were cytarabine and idarubicin, which is an anthracycline. Cytarabine is an antimetabolic agent that causes DNA damage when the cell cycle holds in the S-phase [35]. Anthracycline kills leukemia cells via 3 mechanisms: inhibiting DNA and RNA synthesis by intercalating between base pairs of the DNA/RNA strand; inhibiting topoisomerase II enzyme, preventing the relaxation of supercoiled DNA, and thus blocking DNA transcription and replication; and creating iron-mediated free oxygen radicals that damage DNA and cell membranes [36]. In our study, *BDH2*-KD cells were more sensitive to ROS stimulation and more susceptible to apoptosis than parental and shRNA control vector transfected cells. Among the regulators of programmed cell death, or apoptosis, members of the Bcl-2 family control the release of apoptogenic proteins from mitochondria [37], whereas members of the IAP gene family act as endogenous inhibitors of caspases [38]. No difference was observed between Bcl-2 and Bcl-XL expression in parental, control vector, and *BDH2*-KD leukemia cell lines, before or after H_2_O_2_ treatment. The data showed that survivin was less in *BDH2*-KD cells than control cells, after ROS stimulation, but did not change before ROS stimulation. The other IAP, XIAP, did not differ significantly between *BDH2*-KD and control cells, before or after ROS stimulation. We also observed a limited increase in the cleaved form of caspase-3 in *BDH2*-KD cells by flow cytometry analysis. Survivin, the smallest member of the IAP family, has a synergic effect with XIAP in cytoprotection [39]. Survivin inhibits active caspase-9, but not active caspase-3 [40]. Survivin is selectively expressed in most human cancers, including lung, breast, pancreatic, and colon carcinomas; soft tissue sarcomas; brain tumors; melanoma; neuroblastoma; and hematologic malignancies [40]. We observed that *BDH2*-KD cells had a higher apoptotic rate under ROS stimulation, mediated by suppression of survivin release after ROS exposure.

Here, we showed that *BDH2* overexpression could shorten overall survival and decrease the response rate from intensive induction chemotherapy. The mechanism by which *BDH2* works as an anti-apoptotic factor is mediated by survivin through a caspase-3 independent pathway. To the best of our knowledge, we are the first to report that *BDH2* is newly identified as a poor independent prognostic factor for CN-AML. Lower responsiveness to chemotherapy is predicted and hematological stem cell transplantation should be considered for patients with high expression of *BDH2*. The limitations of our work are related small patients’ number and not purify leukemia or CD34 (+) hematopoietic cells while collected bone marrow samples. We did not find difference of CD34 content in bone marrow samples between *BDH2* higher and lower expression groups. On the other hand, we performed a study to induce THP1 to mature monocytes by using 1,25 Vitamine D3, and detect the *BDH2* mRNA expression level. We did not find difference in native THP1 cells and mature treated THP1 cells (data not shown) [23].

In our future work, we will enroll more AML patients, especially patients receiving hematological stem cell transplantation to analysis the impact of *BDH2*. We will prospectively collect bone marrow from AML patients and sort CD34+ hematopoietic cells and perform those experiments for analysis prognosis. On the other hand, since *BDH2* is an anti-apoptosis factor, we will analyze its impact with other factors which are related to apoptosis, like *Wilms tumor 1*[41], and *DNA methyltransferase 3*[42].

## Conclusion

We reported that *BDH2* overexpression could shorten overall survival and decrease the response rate from intensive induction chemotherapy. The mechanism by which BDH2 works as an anti-apoptotic factor is mediated by survivin through a caspase-3 independent pathway.

## Abbreviations

BDH2: Type 2-hydroxybutyrate dehydrogenase; LCN2: Lipocalin-2; CEBPA: CCAAT/enhancer binding protein (C/EBP), alpha; FLT3-ITD: Fms-related tyrosine kinase 3 Internal tandem duplications; NPM1: Nucleophosmin 1; IDH1/2: Isocitrate dehydrogenase 1/2; DNMT3A: DNA (cytosine-5)-methyltransferase 3A; MLL: Mixed lineage leukemia gene; MN1: Meningionma1; ERG: ETS-related gene; miR-181a: Micro-RNA-181a; miR-3151: Micro-RNA-3151; CR: Complete remission; OS: Overall survival; RFS: Relapse free survival; LFS: Leukemia free survival.

## Competing interests

The authors declare no competing financial interests.

## Authors’ contributions

WC designed and performed research experiments, analyzed data, and wrote the manuscript, WT and PL performed some research experiments, MY provided cells and reagents, and reviewed and edited the manuscript, YL collected patient data and samples, CC provided suggestions to the research and data analysis, WY performed research experiments, SL reviewed and edited the manuscript, provided suggestions to the research. All authors read and approved the final manuscript.

## Supplementary Material

Additional file 1: Figure S1 mRNA expression levels of *BDH2* in bone marrow. *BDH2*mRNA expression levels in bone marrow from patients with normal bone marrow, cytogenetically normal AML (CN-AML), good risk patients with AML-ETO fusion gene and chromosome inv (16), and poor risk patients with multiple chromosome abnormalities. *P<0.001.* Higher ΔC_T-*BDH2*_ means lower mRNA expression levels. BM: bone marrow. Sample number of bone marrows from normal bone marrow, CN-AML, AML with AML-ETO (+), AML with inv (16), and AML with multiple chromosome abnormalities are 43, 113, 10, 3 and 25. Click here for file

Additional file 2: Figure S2 Correlation between *BDH2* and *LCN2* mRNA expression in bone marrow. There is no correlation between the mRNA expression of *BDH2* and *LCN2*. Click here for file

Additional file 3: Figure S3 The Kaplan-Meier overall survival curves in different *BDH2* expression groups. HSCT indicates hematopoietic stem cell transplantation. Click here for file

Additional file 4: Figure S4 Apoptosis after 50 μM H_2_O_2_ treatment for 2 h in each cell line, assessed under a light microscope at 200×. Blue arrows indicate apoptotic cells. shRNA-*BDH2-3* HL60 cells had more apoptotic cells. Click here for file

Additional file 5: Figure S5 The mitochondrial membrane potential change. That was analyzed using a MitoProbe™ JC-1 Assay Kit and no difference between *BDH2*-knockdown THP1 cells and control cells. shRNA1, shRNA-*BDH2*-1 THP1; shRNA3, shRNA-*BDH2*-3 THP1; shRNAc, shRNA empty vector infected THP1; CCCP, carbonyl cyanide 3-chlorophenylhydrazone, positive control. Click here for file
