# Supplementary figures and images for: Human BDH2, an anti-apoptosis factor, is a novel poor prognostic factor for de novo cytogenetically normal acute myeloid leukemia
Source: J Biomed Sci. 2013 Aug 14;20(1):58. doi: 10.1186/1423-0127-20-58 (PMC3844465; doi:10.1186/1423-0127-20-58)

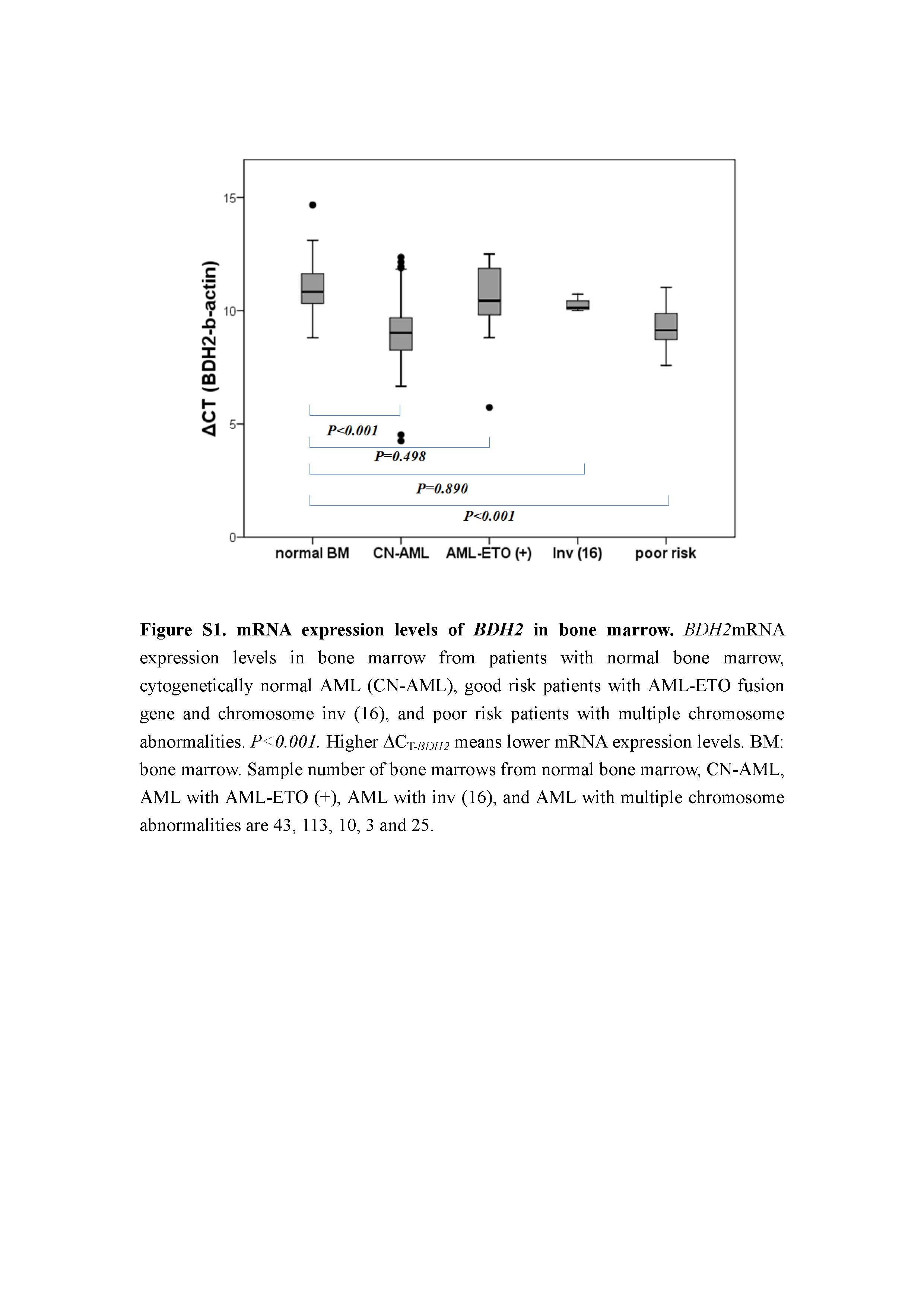

Supplement: Additional file 1: Figure S1 — mRNA expression levels of BDH2 in bone marrow. BDH2mRNA expression levels in bone marrow from patients with normal bone marrow, cytogenetically normal AML (CN-AML), good risk patients with AML-ETO fusion gene and chromosome inv (16), and poor risk patients with multiple chromosome abnormalities. P<0.001. Higher ΔCT-BDH2 means lower mRNA expression levels. BM: bone marrow. Sample number of bone marrows from normal bone marrow, CN-AML, AML with AML-ETO (+), AML with inv (16), and AML with multiple chromosome abnormalities are 43, 113, 10, 3 and 25. [file 1423-0127-20-58-S1.tiff]

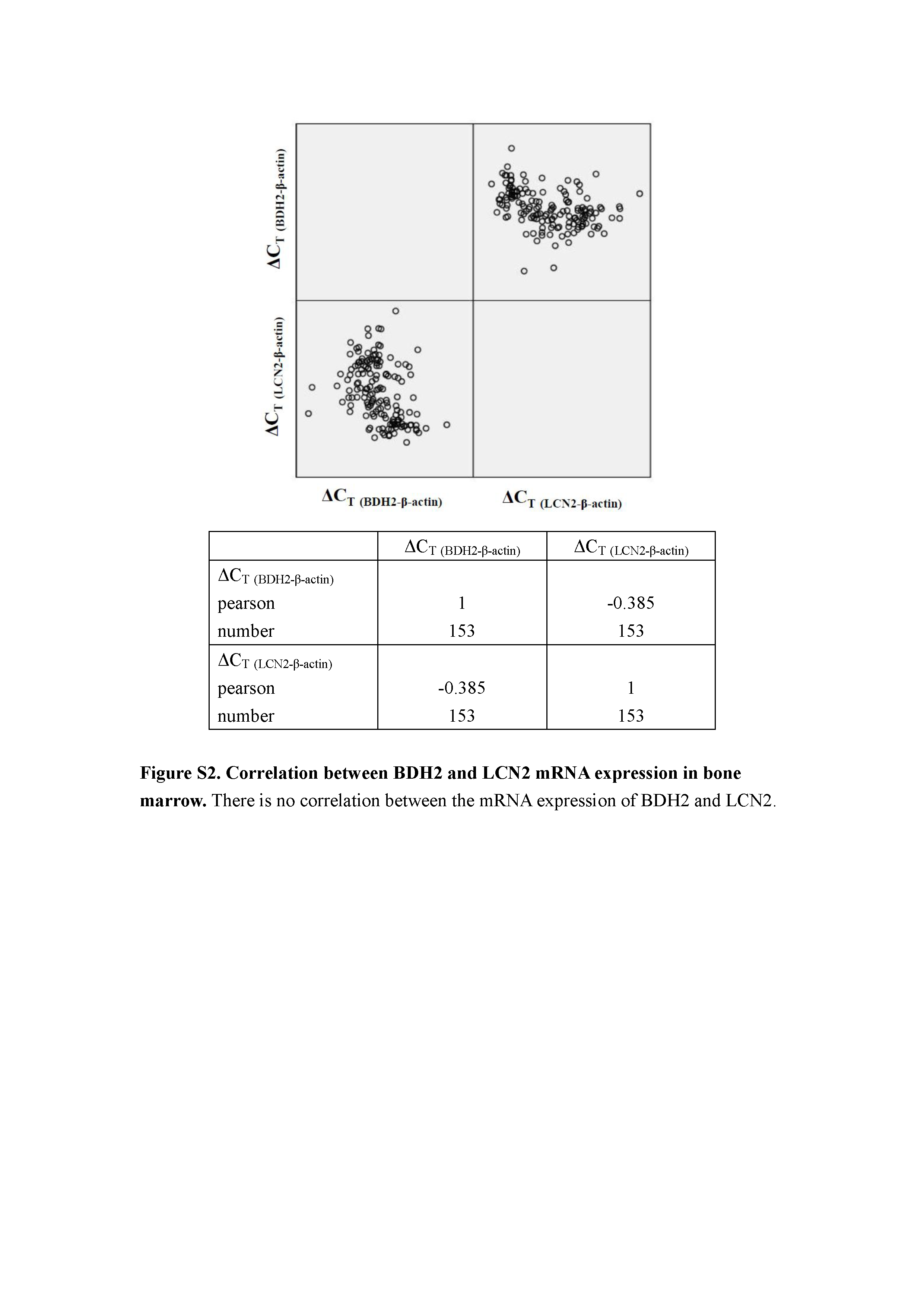

Supplement: Additional file 2: Figure S2 — Correlation between BDH2 and LCN2 mRNA expression in bone marrow. There is no correlation between the mRNA expression of BDH2 and LCN2. [file 1423-0127-20-58-S2.tiff]

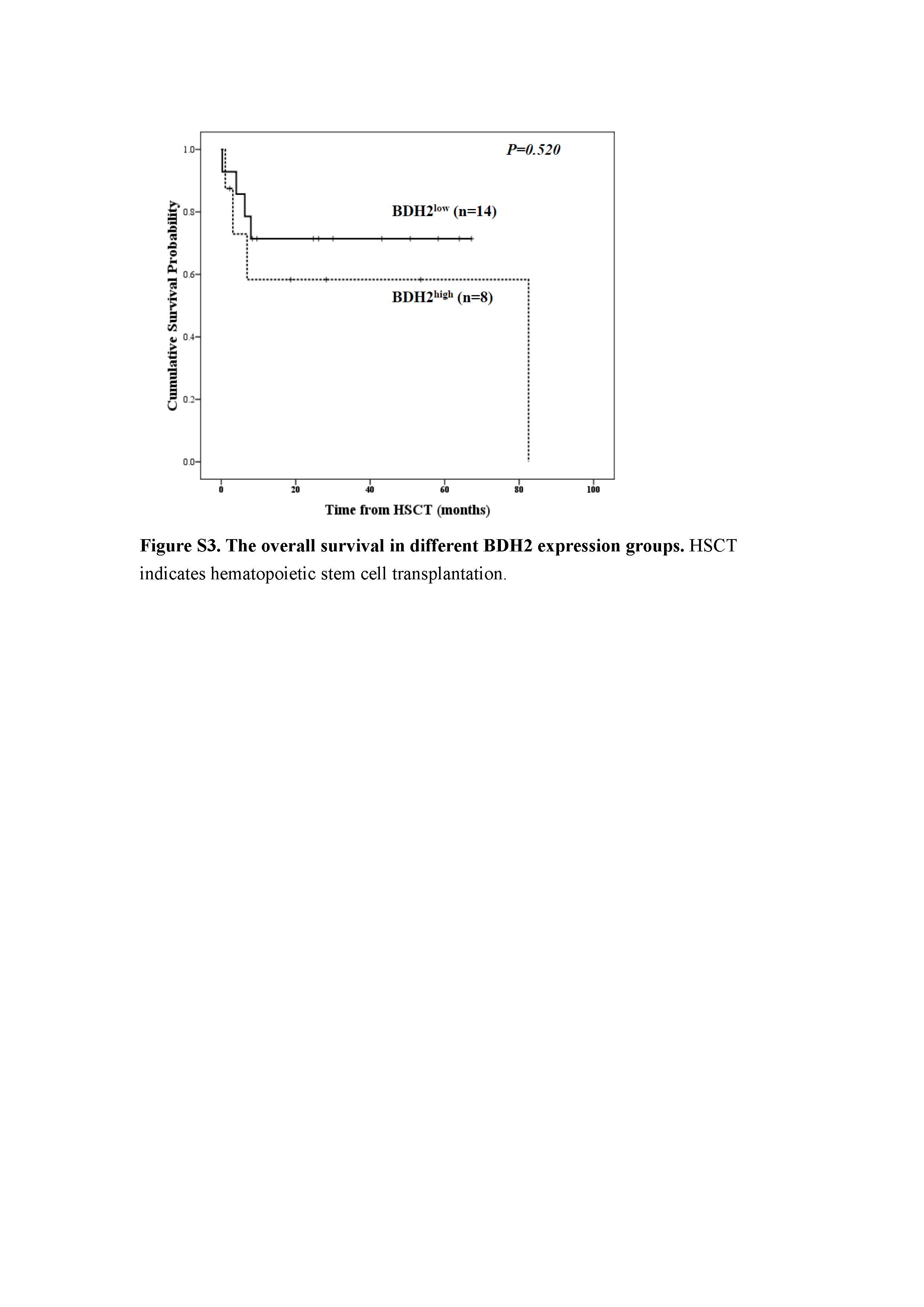

Supplement: Additional file 3: Figure S3 — The Kaplan-Meier overall survival curves in different BDH2 expression groups. HSCT indicates hematopoietic stem cell transplantation. [file 1423-0127-20-58-S3.tiff]

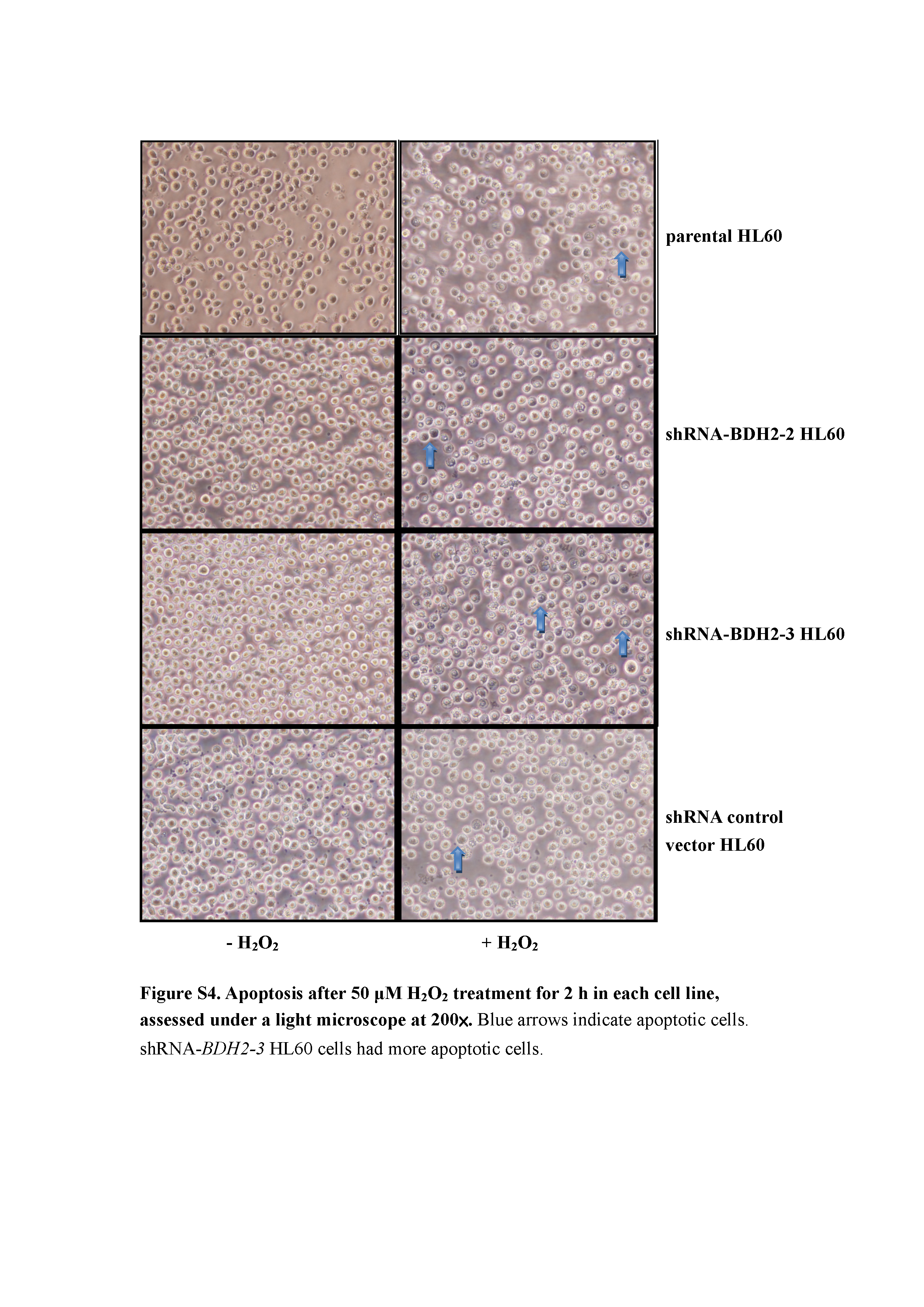

Supplement: Additional file 4: Figure S4 — Apoptosis after 50 μM H2O2 treatment for 2 h in each cell line, assessed under a light microscope at 200×. Blue arrows indicate apoptotic cells. shRNA-BDH2-3 HL60 cells had more apoptotic cells. [file 1423-0127-20-58-S4.tiff]

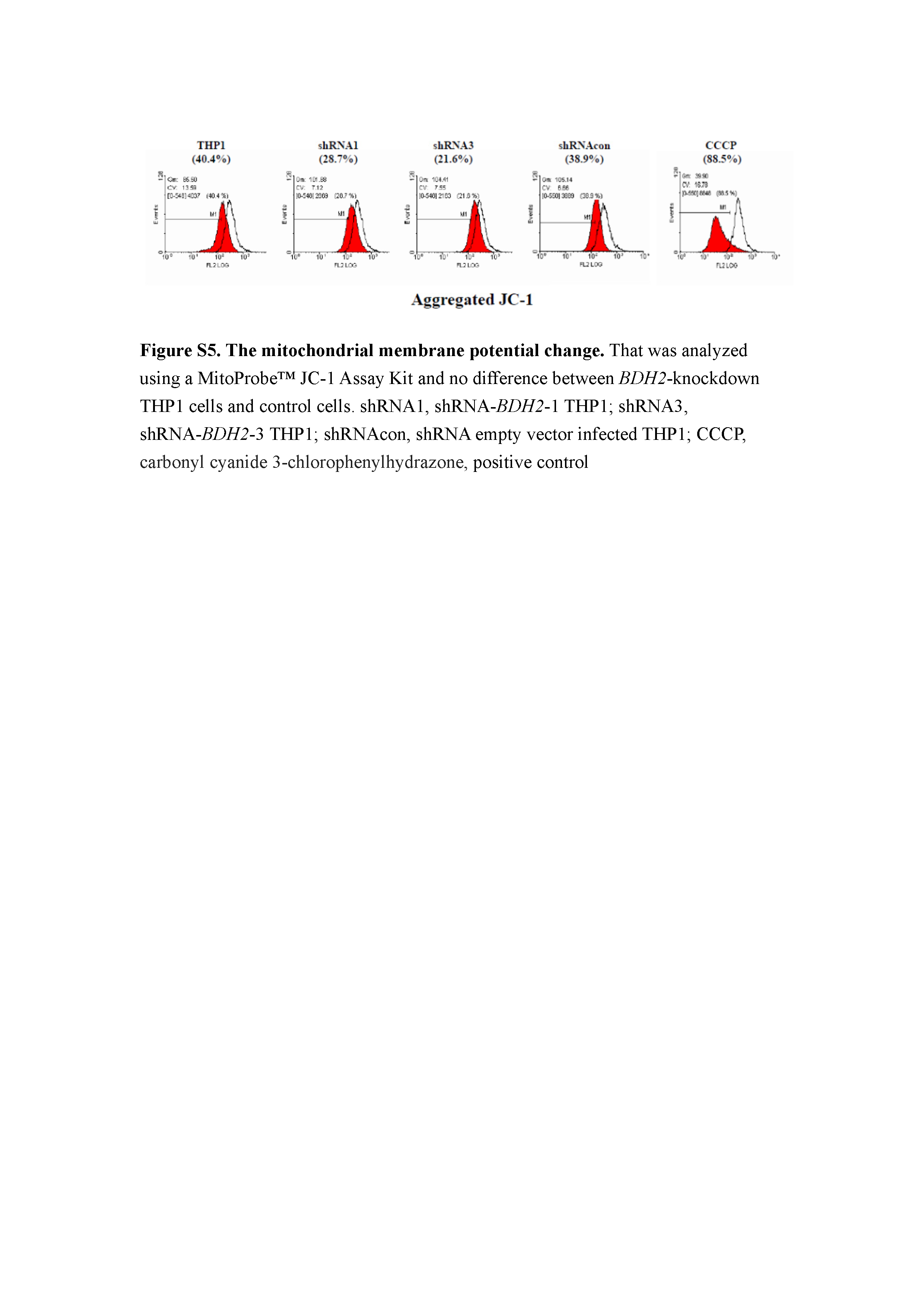

Supplement: Additional file 5: Figure S5 — The mitochondrial membrane potential change. That was analyzed using a MitoProbe™ JC-1 Assay Kit and no difference between BDH2-knockdown THP1 cells and control cells. shRNA1, shRNA-BDH2-1 THP1; shRNA3, shRNA-BDH2-3 THP1; shRNAc, shRNA empty vector infected THP1; CCCP, carbonyl cyanide 3-chlorophenylhydrazone, positive control. [file 1423-0127-20-58-S5.tiff]
